# Protein Tyrosine Phosphatase Non-Receptor Type 22 Modulates NOD2-Induced Cytokine Release and Autophagy

**DOI:** 10.1371/journal.pone.0072384

**Published:** 2013-08-26

**Authors:** Marianne R. Spalinger, Silvia Lang, Stephan R. Vavricka, Michael Fried, Gerhard Rogler, Michael Scharl

**Affiliations:** 1 Division of Gastroenterology and Hepatology, University Hospital Zurich, Zurich, Switzerland; 2 Zurich Center for Integrative Human Physiology, University of Zurich, Zurich, Switzerland; 3 Division of Gastroenterology and Hepatology, Stadtspital Triemli, Zurich, Switzerland; University of Cologne, Germany

## Abstract

**Background:**

Variations within the gene locus encoding protein tyrosine phosphatase non-receptor type 22 (PTPN22) are associated with the risk to develop inflammatory bowel disease (IBD). PTPN22 is involved in the regulation of T- and B-cell receptor signaling, but although it is highly expressed in innate immune cells, its function in other signaling pathways is less clear. Here, we study whether loss of PTPN22 controls muramyl-dipeptide (MDP)-induced signaling and effects in immune cells.

**Material & Methods:**

Stable knockdown of PTPN22 was induced in THP-1 cells by shRNA transduction prior to stimulation with the NOD2 ligand MDP. Cells were analyzed for signaling protein activation and mRNA expression by Western blot and quantitative PCR; cytokine secretion was assessed by ELISA, autophagosome induction by Western blot and immunofluorescence staining. Bone marrow derived dendritic cells (BMDC) were obtained from PTPN22 knockout mice or wild-type animals.

**Results:**

MDP-treatment induced PTPN22 expression and activity in human and mouse cells. Knockdown of PTPN22 enhanced MDP-induced activation of mitogen-activated protein kinase (MAPK)-isoforms p38 and c-Jun N-terminal kinase as well as canonical NF-κB signaling molecules in THP-1 cells and BMDC derived from PTPN22 knockout mice. Loss of PTPN22 enhanced mRNA levels and secretion of interleukin (IL)-6, IL-8 and TNF in THP-1 cells and PTPN22 knockout BMDC. Additionally, loss of PTPN22 resulted in increased, MDP-mediated autophagy in human and mouse cells.

**Conclusions:**

Our data demonstrate that PTPN22 controls NOD2 signaling, and loss of PTPN22 renders monocytes more reactive towards bacterial products, what might explain the association of PTPN22 variants with IBD pathogenesis.

## Introduction

In the healthy gut, commensal bacteria populate our intestine without provoking a significant immune reaction. However, there is an intense and effective immune response as soon as pathogenic bacteria penetrate the epithelial surface. Usually, monocytes and macrophages initiate and orchestrate effective immune responses by the secretion of pro-inflammatory cytokines, as soon pathogenic bacterial components are present. When recruited to the intestine however, peripheral blood monocytes (PBMC) differentiate into intestinal macrophages (iMAC) that are characterized by reduced reactivity and immune tolerance towards commensal bacteria [1]. If the discrimination between pathogenic and commensal bacteria in the gut is disturbed, tolerance is lost and hyper-activated intestinal macrophages drive intestinal inflammation, ultimately resulting in chronic inflammatory conditions as can be observed during inflammatory bowel diseases (IBD) with its major subforms, ulcerative colitis (UC) and Crohn’s disease (CD).

Monocytes, like other innate immune cells, sense bacteria via conserved pattern recognition receptors (PRR), including nucleotide-binding and oligomerization domain containing type 2 (NOD2) [2]. It is obvious that a very tight control of PRR activation is crucial for tolerance towards commensal gut flora. NOD2 is a cytosolic receptor that recognizes invading bacteria by ligation to muramyl-dipeptide (MDP) [3], a highly conserved bacterial cell wall component. Presence of certain polymorphisms within the gene encoding NOD2, that result in aberrant receptor activation, are associated with IBD [4] [5], and malfunction in NOD2 receptor activation interferes with effective clearance of intracellular bacteria in the gut [6].

Upon activation, NOD2 induces the phosphorylation of proteins of the nuclear factor κB (NF-κB) and mitogen-activated protein kinase (MAPK) signaling pathways, resulting in enhanced expression of adhesions molecules and the secretion of pro-inflammatory cytokines [7]. Additionally, NOD2 ligation leads to the induction of autophagy [8]. Autophagy is a homeostatic process involved in removal of damaged proteins and organelles in the cytosol, but it also plays an important role in host defense and clearance of intracellular bacteria [9]. Changes in autophagy are involved in IBD pathogenesis, and variants in autophagy-16 like 1 (ATG16L1), a protein crucial for autophagosome formation, result in an enhanced risk for developing CD [10].

Genome-wide association studies revealed that variants within the gene locus encoding for protein tyrosine phosphatase non-receptor type 22 (PTPN22) are linked with the risk to develop autoimmune disorders, including rheumatoid arthritis, type 1 diabetes, UC and CD [11]. Yet, the functional link between the presence of PTPN22 variants and inflammatory diseases is still not well understood. By dephosphorylation of signaling molecules, tyrosine phosphatases are generally involved in the regulation of immune receptor activity. PTPN22 in particular, has been shown to negatively regulate signaling molecules downstream of T- and B-cell receptors [12], [13] and disease associated variants lead to altered B-cell, NK-cell and dendritic cell (DC) activation [13]–[15]. However it has not been addressed if PTPN22 also interferes with pattern recognition receptor induced signaling cascades. We have previously shown that PTPN22 expression is decreased in the intestine of patients with active CD, in particular in CD68-positive monocytes/macrophages. Loss of PTPN22 results in decreased signal transducer and activator of transcription (STAT)-1, but increased MAPK-signaling in human monocytes. On a functional level, PTPN22-deficiency results in elevated levels of interleukin (IL)-6 and IL-17 secretion [16].

Here, we address the question whether PTPN22 affects NOD2-induced signaling pathways, cytokine secretion, and autophagy in myeloid cells. We found that loss of PTPN22 leads to aberrant MDP/NOD2-induced MAPK and NF-κB) activation, resulting in changed gene expression and cytokine secretion. Additionally, MDP-induced autophagy was enhanced when PTPN22 was missing. This influence of PTPN22 on IBD associated intracellular pathways could provide an explanation of how PTPN22 variants might functionally be linked with IBD.

## Materials and Methods

### Reagents and Antibodies

All reagents were of analytical grade and purchased commercially. Monoclonal goat anti-PTPN22 antibody was obtained from Santa Cruz Biotechnologies (Santa Cruz, CA) and mouse anti-β-actin antibody from Millipore (Billerica, MA). Mouse anti-phospho-p38 (Thr^180^/Tyr^182^), rabbit anti-p38, rabbit anti-phospho-extracellular signal-regulated kinase (ERK)1/2 (Tyr^42^/Tyr^44^), rabbit anti-ERK1/2, mouse anti-phospho c-Jun N-terminal kinase (JNK; Thr^183^/Tyr^185^), rabbit anti-JNK, rabbit anti-phospho-NF-κB-p65 (Ser^536^), rabbit anti-NF-κB-p65 (ser^276^), rabbit anti-phospho-NF-κB p105 (Ser^933^), rabbit anti-NF-κB p105/p50, rabbit anti-phospho-NF-κB p100/p52 (Ser^866^/Ser^870^), rabbit anti-NF-κB p100/p52, and mouse anti-microtubule-associated proteins 1A/1B light chain 3B (LC3B) were obtained from Cell Signaling Technologies (Danvers, MA).

### Cell Culture and Vector Transduction

THP-1 monocytes were cultured in RPMI 1640 (Invitrogen, Carlsbad, CA) supplemented with 10% FCS at a density between 0.2 and 1×10^6^ cells/ml. For experiments, cells were seed in 24 well plates at a density of 0.5×10^6^ cells/ml 24 h before treatment. To generate THP-1 cells stably expressing either non-targeting control or PTPN22 specific shRNA, the following vectors were obtained from Sigma: pLKO.1 (control vector) and pLKO.1-shPTPN22 (NM_008979.1-1233s1c1, PTPN22 targeting vecor), pMD2.G (packaging plasmid) and pCMV (envelop plasmid). Transductions were performed as described elsewhere [17] and cells cultured in RPMI 1640 supplemented with 10% FCS and 20 ng/ml puromycin (Invivogen, San Diego, CA). Stability of the knockdown was determined prior to each experiment by real-time PCR and knockdown efficiency found between 20–40%. In addition knockdown was verified after each experiment by Western blot or real-time PCR. For NOD2 activation, MDP (Invivogen) was dissolved in DMSO and mixed with FuGene (Promega, Fitchburg, WI) in culture media for 15 min before applying on the cells. For control transfections, DMSO mixed with FuGene was used.

### Generation of Bone Marrow Dendritic Cells (BMDC)

Animal experiments were carried out according to Swiss animal welfare laws and were approved by the veterinary authorities of Zurich, Switzerland (Kanton Zürich Gesundheitsdirektion Veterinäramt, approval no. 54/2011). Due to the approval of the veterinary authorities of Zürich, no further approval by an Institutional Animal Care and Use Committee (IACUC) or ethics committee was necessary. PTPN22 knockout mice were kindly provided by Andrew C. Chan, Genentech, Inc., South San Francisco, CA [18]. Bone marrow was isolated from 4–6 old WT or PTPN22 KO mice by flushing femora and tibiae and single cell suspensions prepared using a 26 G needle. After centrifugation (10 min, 320 g), cells were suspended in differentiation medium (RPMI, 10% FCS, 1000 U/ml mouse GM-CSF) and passed through a 70 µm nylon mesh before plating in 6 well plates for seven days. Medium was replaced with fresh differentiation medium at days 3. After 7 days, adhering cells were harvested, 1×10^6^ cells/well seeded in 6 well plates and left to adhere to the plates for 24 h before performing experiments.

### Lysate Preparation

Cells were washed twice with ice-cold phosphate buffered saline (PBS) and lysed in M-Per Mammalian protein extraction reagent (Pierce Biotechnology, Rockford, IL) supplemented with protease inhibitors (Roche, Basel, Switzerland) for 45 min. Cells were centrifuged for 10 min at 13,000 g and supernatants assayed for protein content by absorbance measurement (NanoDrop ND1000; Pierce Biotechnology).

### Western Blotting

An aliquot of each lysate was mixed with loading buffer (NuPAGE® 4×LDS Sample Buffer (Invitrogen), 50 mM dithiothreitol) and boiled for 5 min at 96°C. Proteins were separated by SDS-polyacrylamide gel electrophoresis and transferred onto nitrocellulose membranes (GE Healthcare, Little Chalfont, UK). Membranes were blocked with 1% blocking solution and primary antibody (concentrations according to manufactureŕs instructions) was added in blocking buffer (3% BSA in washing buffer (Tris buffered saline containing 1% Tween 20). Membranes were washed for 30 min, HRP-labelled secondary anti-mouse-, anti-goat- or anti-rabbit-IgG-antibody (1∶5000, Santa Cruz) in blocking buffer was added for 30 min and membranes were washed for 30 min. with 1% TBST. Immunoreactive proteins were detected using an enhanced chemiluminescence detection kit (GE Healthcare). Densitometric analysis of Western blots was performed by NIH Image software.

### RNA Isolation and Complementary DNA Synthesis

THP-1 cells were disrupted in RLT buffer (Qiagen) using a 26 G needle. Total RNA was isolated using RNeasy Plus Mini Kit (Qiagen), and DNA removed by TURBO DNA-free Kit (Ambion, Austin, TX) according to manufacturer's instructions. RNA concentration was assessed by absorbance at 260 and 280 nm. Complementary DNA (cDNA) synthesis was performed using a High-Capacity cDNA Reverse Transcription Kit (Applied Biosystems, Foster City, CA) following the manufacturer’s instructions.

### Real-time Polymerase Chain Reaction

Real-time polymerase chain reaction (PCR) was performed using FAST qPCR MasterMix for Taqman Assays (Applied Biosystems) on a Fast HT7900 Real-Time PCR system using SDS Software (Applied Biosystems). Measurements were performed in triplicate, human β-actin was used as endogenous control, and results were analyzed by ΔΔCT method. The real-time PCR contained an initial enzyme activation step (5 min, 95°C) followed by 45 cycles consisting of a denaturing (95°C, 15 seconds) and an annealing/extending (60°, 1 min) step. The used gene expression assays were all obtained from Applied Biosystems.

### Phosphatase Activity Assay

Phosphatase activity was assessed using the EnzChek Phosphatase Assay Kit (Molecular Probes) according to manufacturer's instructions and as described previously [16]. PTPN22 was immunoprecipitated over night from whole cell lysates using anti-PTPN22 antibody (1 µg/mL, Santa Cruz), and fluorescence in immunoprecipitates was detected on a SpectraMax M2 Fluorescence Microplate reader using SoftMax Pro v5 Software (Molecular Devices, Sunnyvale, CA). Measurements were performed in triplicates. Remaining immunoprecipitates were used for Western blotting to ensure equal amounts of PTPN22 in each sample.

### Immunofluorescence Staining

Cells were collected and washed three times in ice cold PBS before transferring to glass slides by cytospin (800 rpm, 5 min). Afterwards, cells were fixed with 4% paraformaldehyde followed by incubation with Methanol at −20°C for 20 min. After 3 wash steps in ice-cold PBS, cells were incubated overnight with anti-LC3 antibody (Cell Signalling, 1∶100). Texas-Red conjugated anti-mouse IgG secondary antibody was added for 2 h, and cell nuclei stained with DAPI for 10 min. Cells were washed 3 times and slides mounted with Fluorescence Mounting Medium (DAKO, Glostrup, Denmark). Cells were imaged immediately using a fluorescence microscope (Zeiss Axio Imager.Z2, Zeiss, Jena, Germany) equipped with an AxioCam HRc (Zeiss) and ApoTome.2 (Zeiss) with AxioVision Release 4.8.2 software (Zeiss).

### Statistical Analysis

Data are presented as means ± S.E.M. for a series of n experiments. Data are expressed as relative values of the respective control. Statistical analysis was performed by analysis of variance (ANOVA) followed by Student–Newman–Keuls post hoc test. P values <0.05 were considered significant.

## Results

### MDP Treatment Induces PTPN22 Expression and Activity

**Figure 1:**
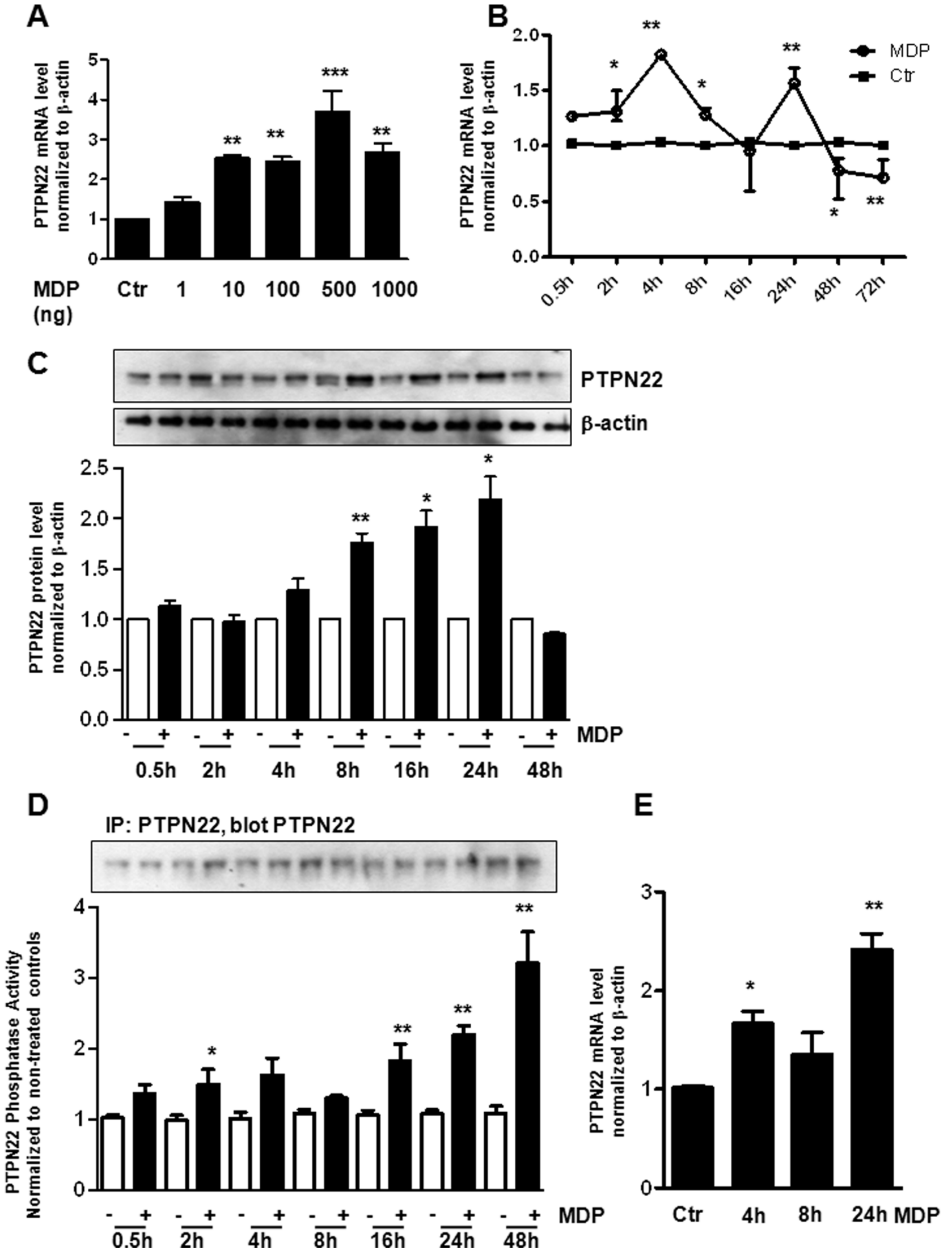
PTPN22 expression and activity is increased by MDP-treatment. (**A**) THP-1 cells were treated with indicated amounts of MDP. The graph depicts relative PTPN22 mRNA levels normalized to β-actin and compared to non-treated controls. (**B+C**) THP-1 cells were treated with MDP (500 ng/ml) for increasing time. (**B**) The graph shows levels of PTPN22 mRNA normalized to β-actin and relative to non-treated control cells. (**C**) Representative Western blots show levels of PTPN22 expression and the loading control β-actin; the graph below depicts results of densitometric analysis (n = 3). (**D**) PTPN22 phosphatase activity normalized to PTPN22 content and relative to untreated controls (n = 3). Western blots above show PTPN22 amounts in the used precipitates. (**E**) BMDCs were treated for the indicated time with 500 ng/ml MDP. The graph shows levels of PTPN22 mRNA normalized to β-actin and relative to non-treated control cells. Asterisks denote significant differences from the respective controls (* = p<0.05, ** = p<0.01, *** = p<0.001).

First, we addressed whether PTPN22 expression in THP-1 cells might be regulated by MDP-induced NOD2 activation. Cells were treated with different concentrations of MDP and a concentration of 500 ng/ml MDP seemed to exert the highest effect on PTPN22 mRNA expression (p<0.001; Figure 1A). Therefore, in our subsequent experiments, cells were treated with 500 ng/ml MDP. We found enhanced levels of PTPN22 mRNA after treatment for 2, 4, 8 and 24 h while longer exposure to MDP (48 h and 72 h) reduced PTPN22 mRNA in THP-1 cells (p<0.01; Figure 1B). On a protein level, we detected enhanced amounts of PTPN22 reaching a maximum by 24 h treatment (p<0.01). After 48 h, PTPN22 levels in MDP-treated cells were comparable to those in untreated cells (Figure 1C). Interestingly, PTPN22 phosphatase activity was increased in response to MDP reaching its maximum only after 48 h (p<0.01; Figure 1D). These observations suggest that expression levels and enzymatic activity of PTPN22 protein are not necessarily correlated. To confirm our findings, we next treated bone marrow derived dendritic cells (BMDC) from wild-type mice for increasing time with MDP. Again, we could detect increased levels of PTPN22 mRNA at 4 h and 24 h (Figure 1E). Taken together these findings demonstrate that MDP induces PTPN22 expression and activity in a time-dependent manner.

To address if activation of other PRRs beside NOD2, might also influence expression of PTPN22, THP-1 cells were treated for increasing time with lipopolysaccharide (LPS, a toll-like receptor (TLR) 4 ligand), the TLR1/2 ligand PamCys or the Nod1 ligand C12-iE-DAP. While stimulation with LPS also led to a significant increase in PTPN22 expression after 24 h, TLR1/2 activation by PamCys reduced PTPN22 expression and C12-iE-DAP-mediated NOD1 activation did not change PTPN22 expression at all (Figures S1A–C). This indicates that PTPN22 is differentially regulated by distinct pathogen associated molecular patterns and suggest that PTPN22 is regulated by the PRR via different signaling pathways.

### Knock-down of PTPN22 Enhances MAPK Activation

**Figure 2:**
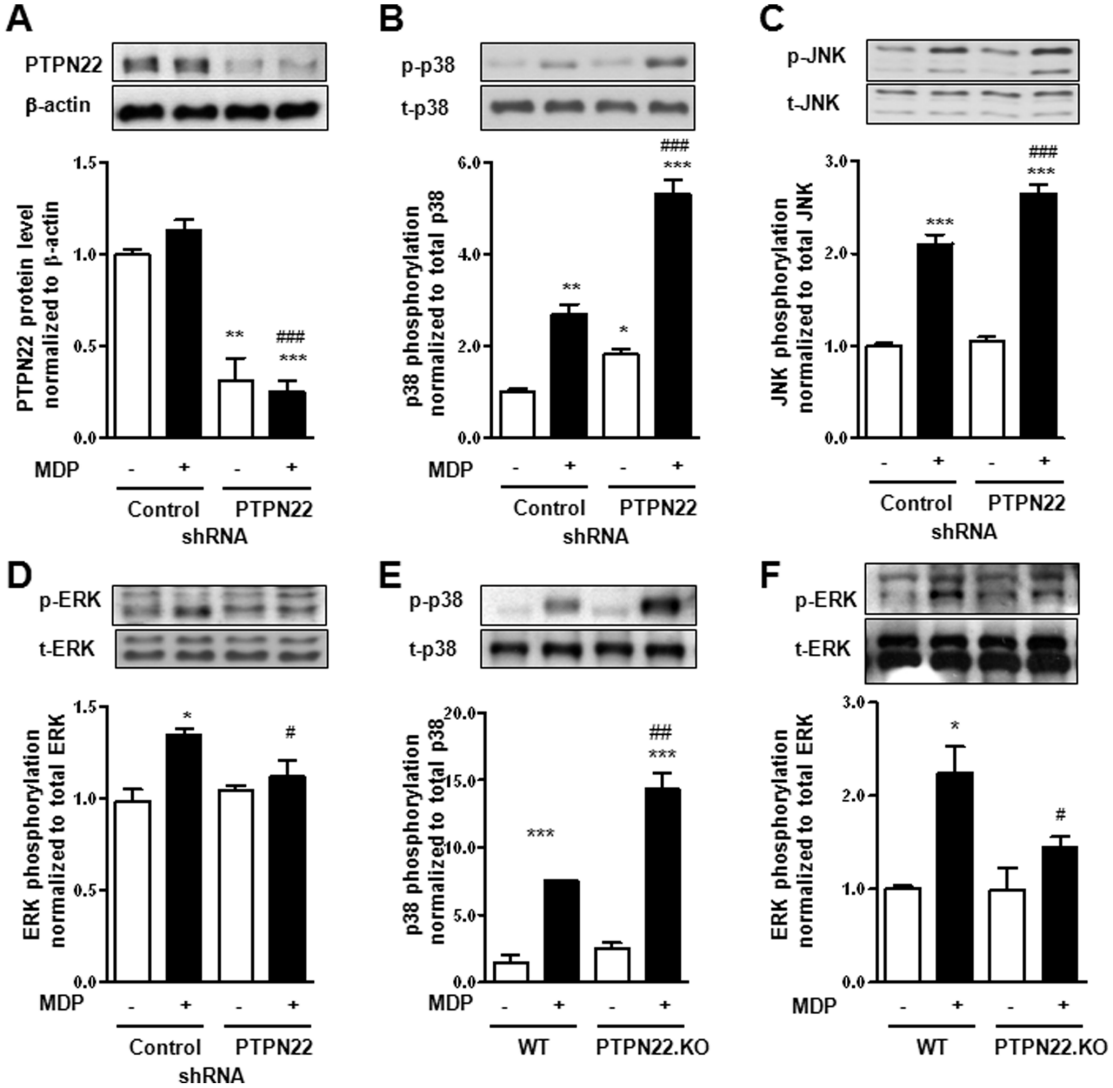
Loss of PTPN22 enhances p38 and JNK MAPK phosphorylation. **A–D:** THP-1 cells, expressing either non-targeting control shRNA or PTPN22 targeting shRNA, were treated for 30 min. with 500 ng/ml MDP. Representative Western blots and respective densitometric analysis show levels of (**A**) PTPN22 and β-actin; (**B**) phospho-p38 (Thr^180^/Tyr^182^) and total p38; (**C**) phospho-JNK (Thr^183^/Tyr^185^) and total JNK; (**D**) phospho-ERK (Tyr^42^/Tyr^44^) and total ERK. (**E+F**) BMDC from either wild type (WT) or PTPN22 knockout (PTPN22.KO) mice were stimulated for 30 min with 500 ng/ml MDP. Representative Western blots and densitometric analysis below show levels of (**E**) phospho-p38 (Thr^180^/Tyr^182^) and total p38; and (**F**) phospho-ERK (Tyr^42^/Tyr^44^) and total ERK. Asterisks denote significant differences from the respective control (n = 3 each, * = p<0.05, ** = p<0.01, *** = p<0.001); # = p<0.05, # = p<0.01, ### = p<0.001 *vs.* MDP-treatment of cells expressing control shRNA.

As PTPN22 is induced by MDP-treatment, we addressed if PTPN22 is involved in the regulation of MDP-induced signaling pathways, such as MAPK or NF-κB activation. For this purpose, THP-1 cells were transduced with lentiviral particles either containing non-targeting control, or PTPN22 silencing shRNA. After selection of cells stably expressing shRNA, PTPN22 expression was reduced for about 60–80% (Figure 2A). This cell lines were used for all subsequent studies. After treatment with MDP, we detected an increase in p38, JNK, and ERK phosphorylation (meaning activation) in control shRNA transduced cells (Figures 2B–D and Figures S2A+B). Loss of PTPN22 enhanced basal and MDP-induced phosphorylation of p38 and MDP-induced JNK phosphorylation. However, ERK activation was delayed and significantly reduced in MDP-treated PTPN22 knockdown cells (Figures 2B–D and Figures S2A+B). To confirm our findings, and rule out possible off-target effects of PTPN22 shRNA, we treated BMDC from either wild type (WT) or PTPN22 knockout (KO) mice for 30 min with MDP. Again, we found increased levels of p38 phosphorylation upon MDP-treatment and this increase was further enhanced in cells lacking PTPN22 (Figure 2E). MDP-induced ERK phosphorylation however was abrogated in PTPN22 KO BMDCs (Figure 2F).

In cell treated with LPS we could find similar effects of PTPN22 knockdown on p38 and ERK activation as in MDP-treated cells (Figure S2C). Interestingly, although stimulation with C12-iE-DAP or PamCys did not lead to an increase in PTPN22 expression, we detected an increased induction of p38 phosphorylation in C12-i-E-DAP or PamCys treated cells lacking PTPN22 (Figures S2D+E). Taken together this indicates that MAPK seem to be regulated by PTPN22 in a stimulus independent way.

### p65 and p105 NF-κB Subunits are Differentially Influenced by PTPN22 Knockdown

**Figure 3:**
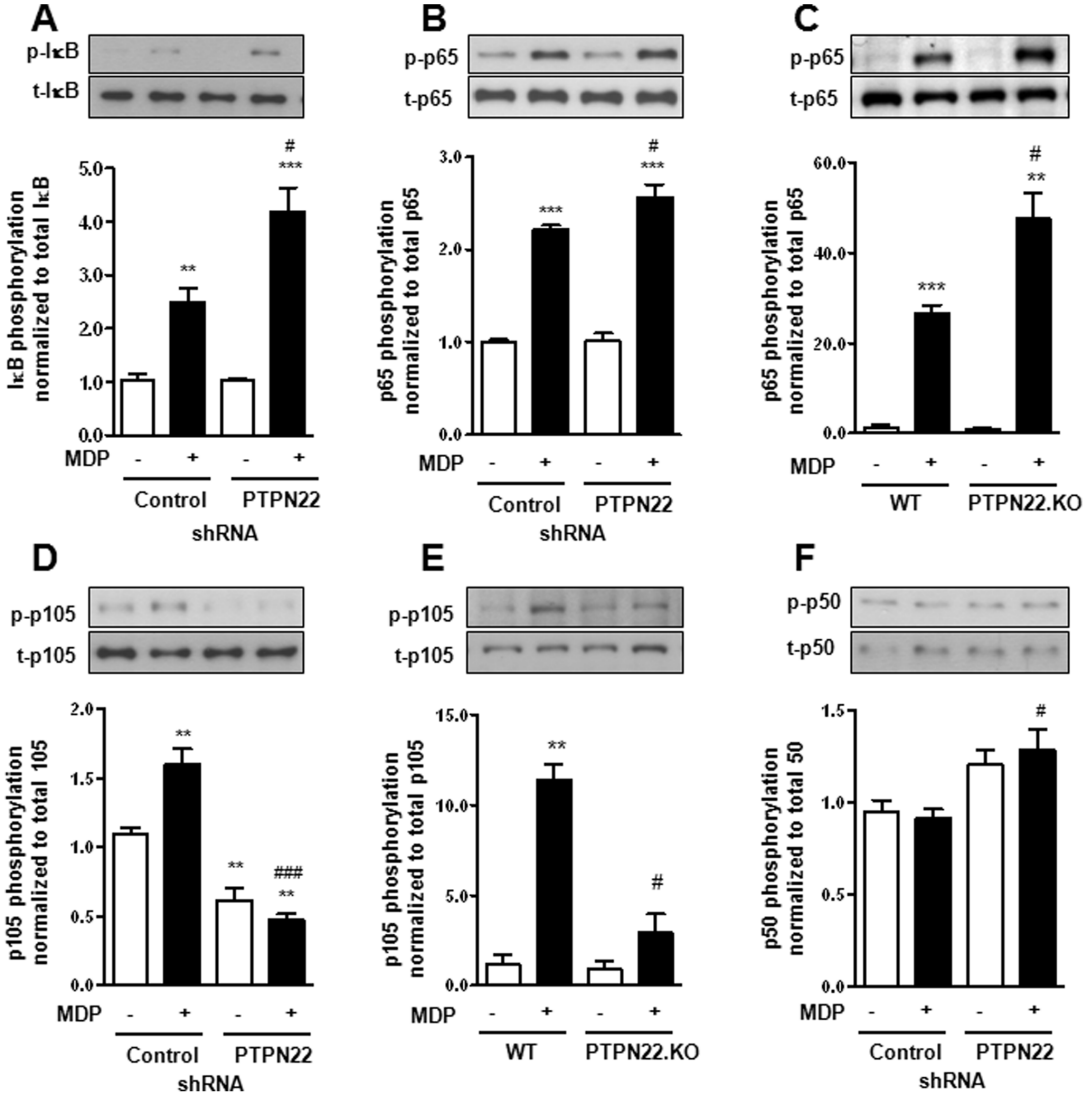
Knockdown of PTPN22 promotes canonical but not non-canonical NF-κB activation. (**A+B**) THP-1 cells, expressing non-targeting control or PTPN22 specific shRNA, were treated for 30 min with 500 ng/ml MDP. Western blots and densitometric analysis below show levels of (**A**) phospho-IκB-α (Ser^32^) and total IκB-α; (**B**) phospho-NF-κB p65 (Ser^536^) and total NK-κB p65. (**C**) BMDC from either wild type (WT) or PTPN22 knockout (PTPN22.KO) mice were treated for 30 min with 500 ng/ml MDP. Representative Western blots and densitometric analysis below show levels of phospho-NF-κB p65 (Ser^536^) and total NK-κB p65. (**D**) THP-1 cells were treated as in A+B, Western blots and densitometric analysis show levels of phospho-NF-κB p105 (Ser^933^) and total NF-κB p105. (**E**) BMDC were treated as in (C); Western blot and densitometric analysis show levels of phospho-NF-κB p105 (Ser^933^) and total NF-κB p105. (**F**) THP-1 cells were treated as in A+B, Western blots and densitometric analysis show levels of phospho-NF-κB p50 (Ser^933^) and total NF-κB p50. Asterisks denote significant differences from the respective control (* = p<0.05, ** = p<0.01, *** = p<0.001); # = p<0.05, ### = p<0.001 *vs.* MDP-treatment of cells expressing control shRNA.

To address the effect of PTPN22 knockdown on NF-κB activation, lysates from MDP-treated cells, expressing either non-targeting control or PTPN22 targeting shRNA, were analyzed for IκB-α and NF-κB p65, p105/50 and p100/52 phosphorylation. MDP induced IκB-α phosphorylation in control-transduced cells, and this induction was enhanced in cells lacking PTPN22 (Figure 3A). MDP induced phosphorylation of NF-κB p65 (RelA) and this effect was further enhanced in PTPN22-deficient THP-1 cells and mouse BMDC (Figures 3B+C, Figure S3A). Additionally, while phosphorylation of the p105 precursor was decreased by knockdown of PTPN22 in human THP-1 cells and mouse BMDC (Figures 3D+E, Figure S3B), phosphorylation of NF-κB p50, which is produced from p105 by constitutive cleavage [19], was enhanced (Figure 3F). In contrast, phosphorylation of NF-κB p100 precursor showed no difference (Figure S3C) by loss of PTPN22. Further, we could not detect altered phosphorylation of the non-canonically activated NF-κB p52 form (Figure S3D) by MDP-treatment or PTPN22 knockdown, and total levels of all addressed NF-κB subunits were unchanged (Figures 3A–F, Figures S3A+D). Interestingly, loss of PTPN22 had different effects on LPS-induced NF-κB activation, where we detected a significant reduction in both, p65 and p105 phosphorylation in PTPN22 deficient cells (Figure S3E). PamCys or C12-iE-DAP mediated NF-κB phosphorylation on the other hand was not significantly affected by loss of PTPN22 (Figures S3F+G). These findings indicate that PTPN22 controls canonical NF-κB, but not non-canonical NF-κB signaling in response to MDP.

### Knock-down of PTPN22 Results in Changes in mRNA Expression and Cytokine Secretion

**Figure 4:**
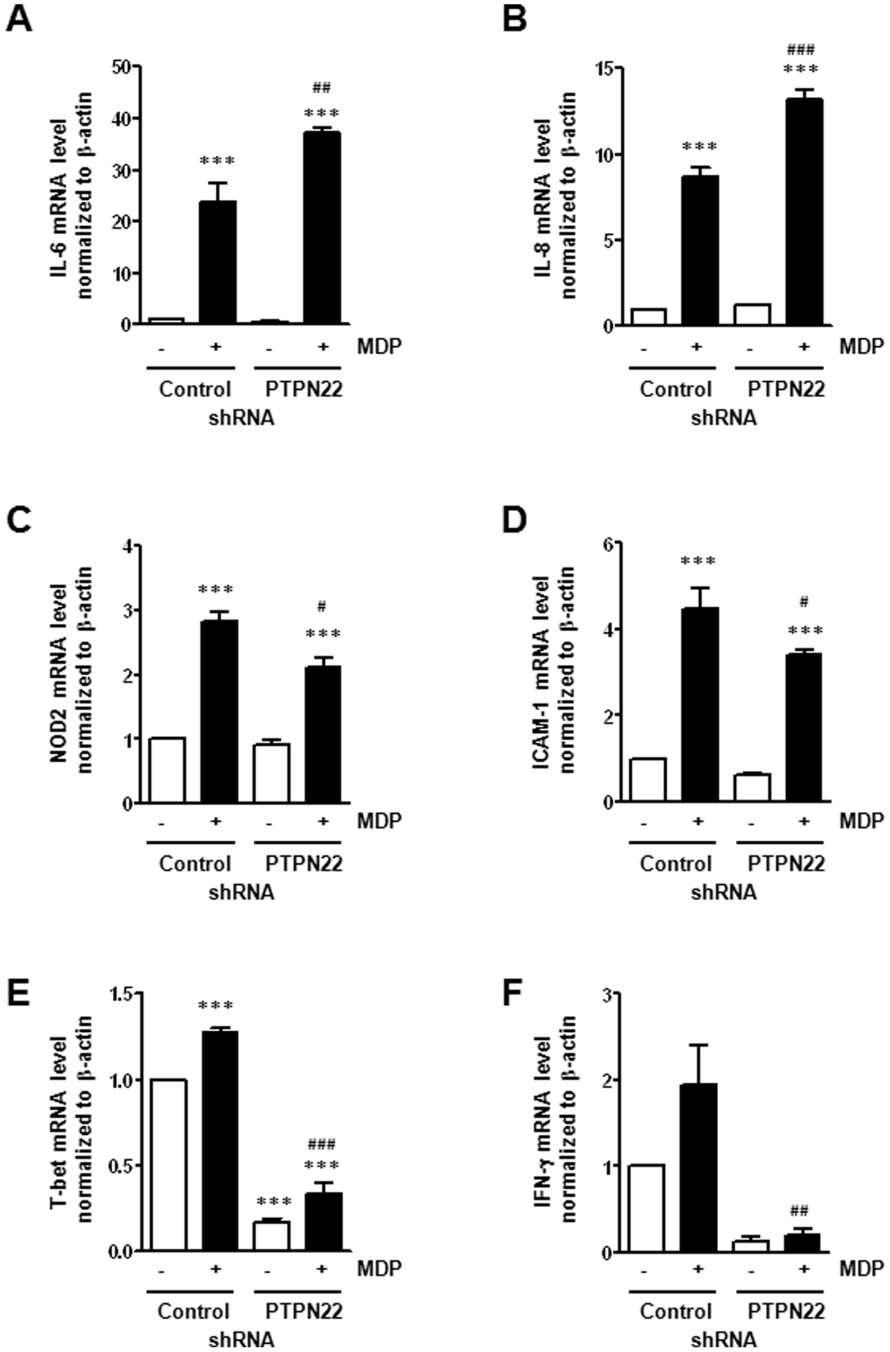
Changed mRNA expression upon knockdown of PTPN22. THP-1 cells, expressing either control or PTPN22 specific shRNA, were treated 24 h with 500 ng/ml MDP. The graphs depict levels of (**A**) IL-6; (**B**) IL-8; (**C**) NOD2; (**D**) ICAM-1; (**E**) T-bet; or (**F**) IFN-γ mRNA normalized to β-actin and relative to non-treated control-shRNA expressing cells. Asterisks denote significant differences from the respective control (*** = p<0.001); # = p<0.05, # = p<0.001, ### = p<0.001 *vs.* MDP-treatment of cells expressing control shRNA.

We next investigated functional consequences arising from the observed alterations in MDP-induced signaling. Consistent with enhanced MAPK and NF-κB p65 activation, we detected increased IL-6 (Figure 4A) and IL-8 mRNA (Figure 4B) expression in 24 h MDP-treated, PTPN22-deficient THP-1 cells, when compared to the respective controls. However, the MDP-induced rise in NOD2 (Figure 4C) and intercellular adhesion molecule 1 (ICAM-1; Figure 4D) mRNA expression was impaired when PTPN22 was missing. Additionally, we detected deceased levels of T-bet mRNA (Figure 4E) and its target gene interferon-γ (IFN-γ; Figure 4F) in untreated and MDP-treated cells.

**Figure 5:**
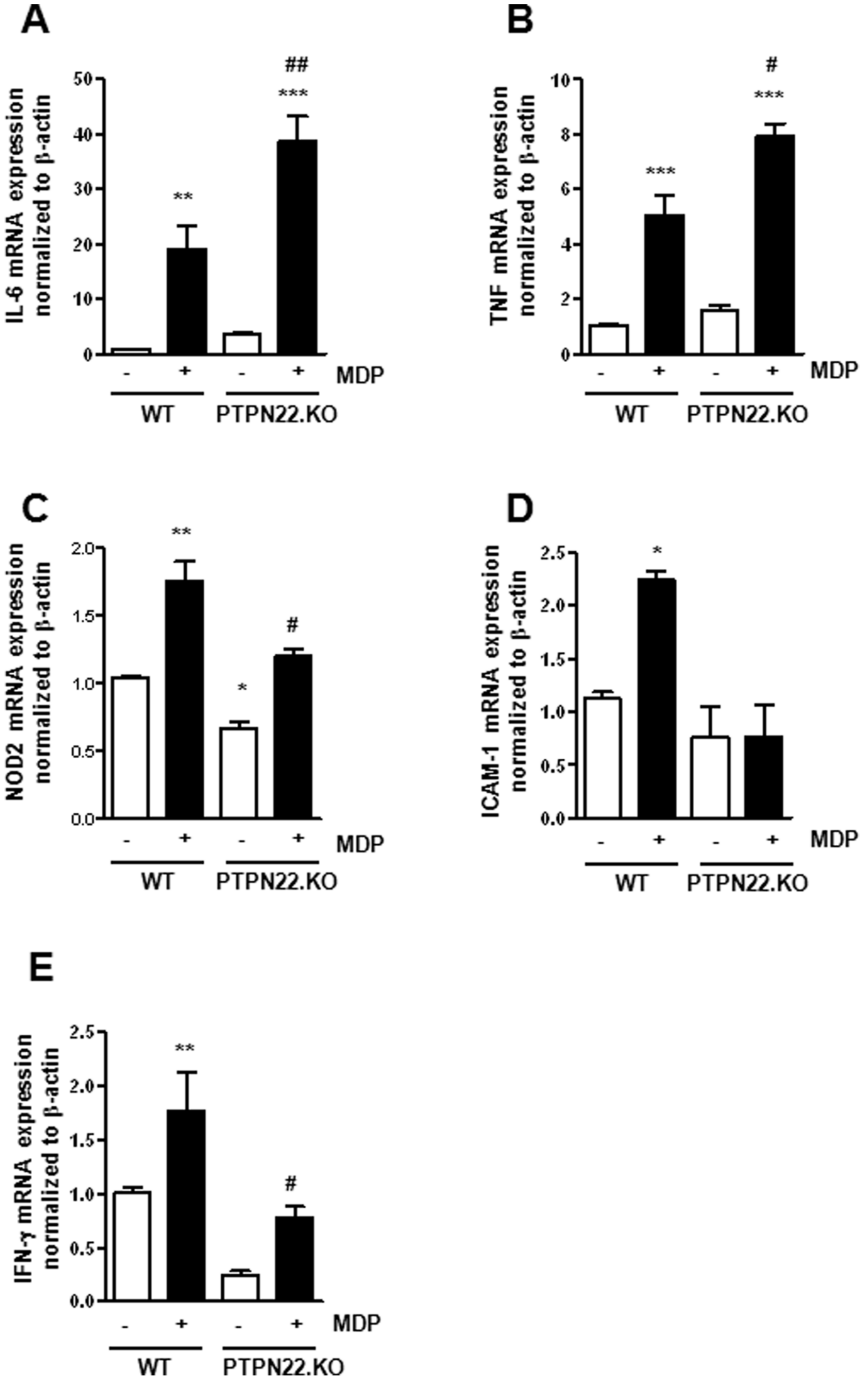
Changed mRNA expression in BMDC lacking PTPN22. BMDC from either wild-type (WT) or PTPN22 knockout (PTPN22.KO) mice, were treated 24 h with 500 ng/ml MDP. The graphs depict levels of (**A**) IL-6; (**B**) TNF; (**C**) NOD2; (**D**) ICAM-1; or (**E**) IFN-γ mRNA normalized to β-actin and relative to non-treated WT BMDC. Asterisks denote significant differences from the respective control (* = p<0.05, ** = p<0.01, *** = p<0.001); # = p<0.05, ## = p<0.001, *vs.* MDP-treatment of WT BMDC.

These findings could be fully confirmed using BMDC derived from PTPN22 knockout mice. Loss of PTPN22 resulted in further increased mRNA levels of IL-6 (Figure 5A) and tumor necrosis factor (TNF; Figure 5B), but decreased mRNA levels of NOD2, ICAM-1 and IFN-γ in BMDC when compared to PTPN22 competent cells from wild-type mice (Figures 5C–E).

**Figure 6:**
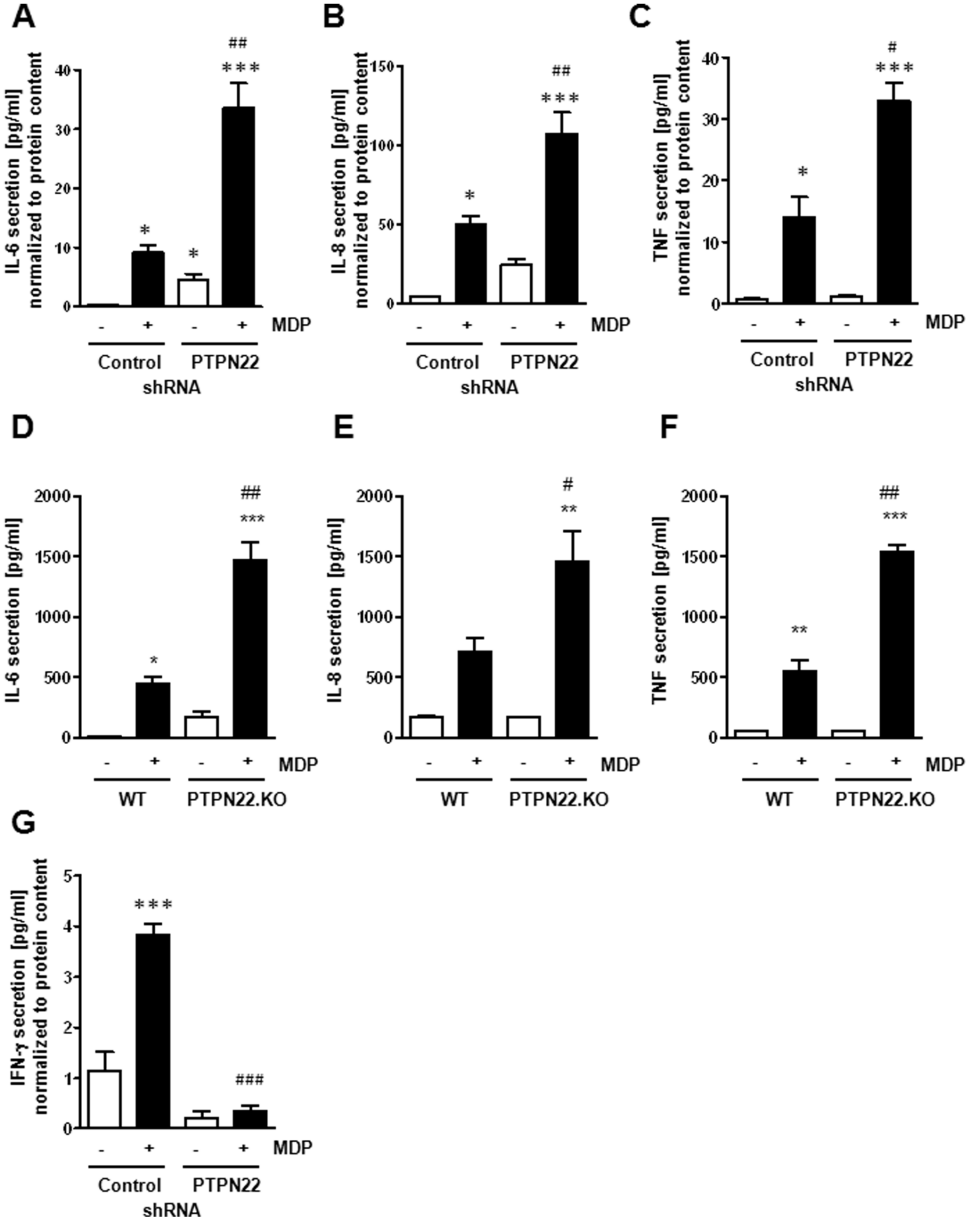
Loss of PTPN22 leads to changes in cytokine secretion. (**A–C**) THP-1 cells, expressing non-targeting control or PTPN22 targeting shRNA, were treated for 24 h with 500 ng/ml MDP. The graphs show the secretion of (**A**) IL-6, (**B**) IL-8; or (**C**) TNF into the cell culture medium. (**D–F**) BMDC from WT or PTPN22 KO mice, were treated for 24 h with 500 ng/ml MDP and analysed for (**D**) IL-6, (**E**) IL-8; or (**F**) TNF secretion into the cell culture medium. (**G**) THP-1 cells were treated as in A–C; the graph shows levels of IFN-γ secretion. Values were shown in pg/ml and normalized to total protein content. Significant differences from the controls are denoted by asterisks (* = p<0.05, ** = p<0.01, *** = p<0.001); # = p<0.05, ## = p<0.01, ### = p<0.001 *vs.* MDP-treated control-transduced cells.

PTPN22 deficiency finally resulted in enhanced secretion of the pro-inflammatory cytokines IL-6, IL-8 and TNF in human THP-1 monocytes (Figures 6A–C) as well as mouse BMDC (Figures 6D–F). Additionally, and consistent with reduced levels of T-bet transcription factor and IFN-γ mRNA, PTPN22 knockdown prevented the MDP-induced rise in IFN-γ secretion in THP-1 monocytes (Figure 6G). These observations demonstrate that PTPN22 controls MDP-induced cytokine secretion and loss of PTPN22 results in an aberrant pattern of cytokine secretion in response to bacterial stimuli.

### Enhanced Autophagy Induction upon Knock-down of PTPN22

**Figure 7:**
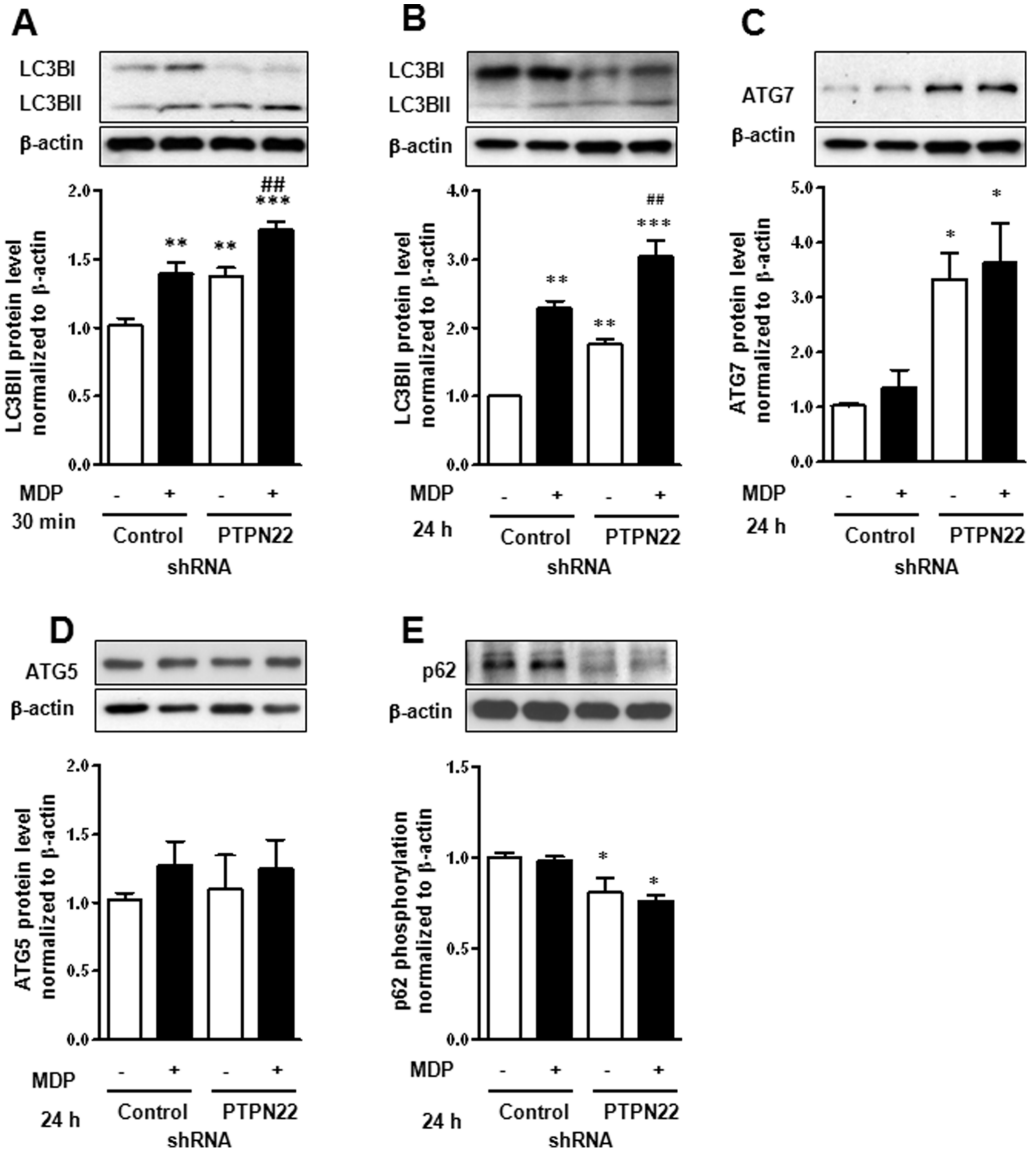
Autophagy is enhanced upon PTPN22 knockdown. THP-1 cells were treated for the indicated time with 500 ng/ml MDP. Western blots and results of densitometric analysis in the graphs below show levels of (**A+B**) LC3BI+II and β-actin; (**C**) ATG7 and β-actin; (**D**) ATG5 and β-actin; or (**E**) p62 and β-actin. Significant differences from the controls are denoted by asterisks (* = p<0.05, ** = p<0.01, *** = p<0.001); ## = p<0.01 *vs.* MDP-treated control-transduced cells.

As NOD2 activation leads to an increase in autophagy [8], we next addressed whether loss of PTPN22 also interferes with autophagosome formation. Therefore, THP-1 cells expressing non-targeting control or PTPN22-silencing shRNA, were treated for 30 min or 24 h with MDP. At both time points, we could detect increased levels of LC3B-II, the cleaved and activated form of LC3B, upon MDP-treatment, indicative for elevated autophagosome formation (Figures 7A+B). In cells transduced with PTPN22 shRNA, LC3B-II levels were increased even prior to MDP-treatment and further enhanced by MDP (Figures 7A+B). No difference in the protein levels of autophagy-like (ATG)5 or ATG7 could be detected in control-transduced MDP-treated cells. In PTPN22 deficient cells however, ATG7 levels were enhanced with or without MDP-treatment, but no change in ATG5 levels could be detected (Figures 7C+D). As p62 transports proteins to the autophagosome and subsequently gets degraded in autolysosomes, its reduction can serve as an additional marker for functional autophagy [20]. Consistent with enhanced LC3B-II levels in PTPN22 deficient cells, we also detected a decrease in p62 protein levels (Figure 7E).

**Figure 8:**
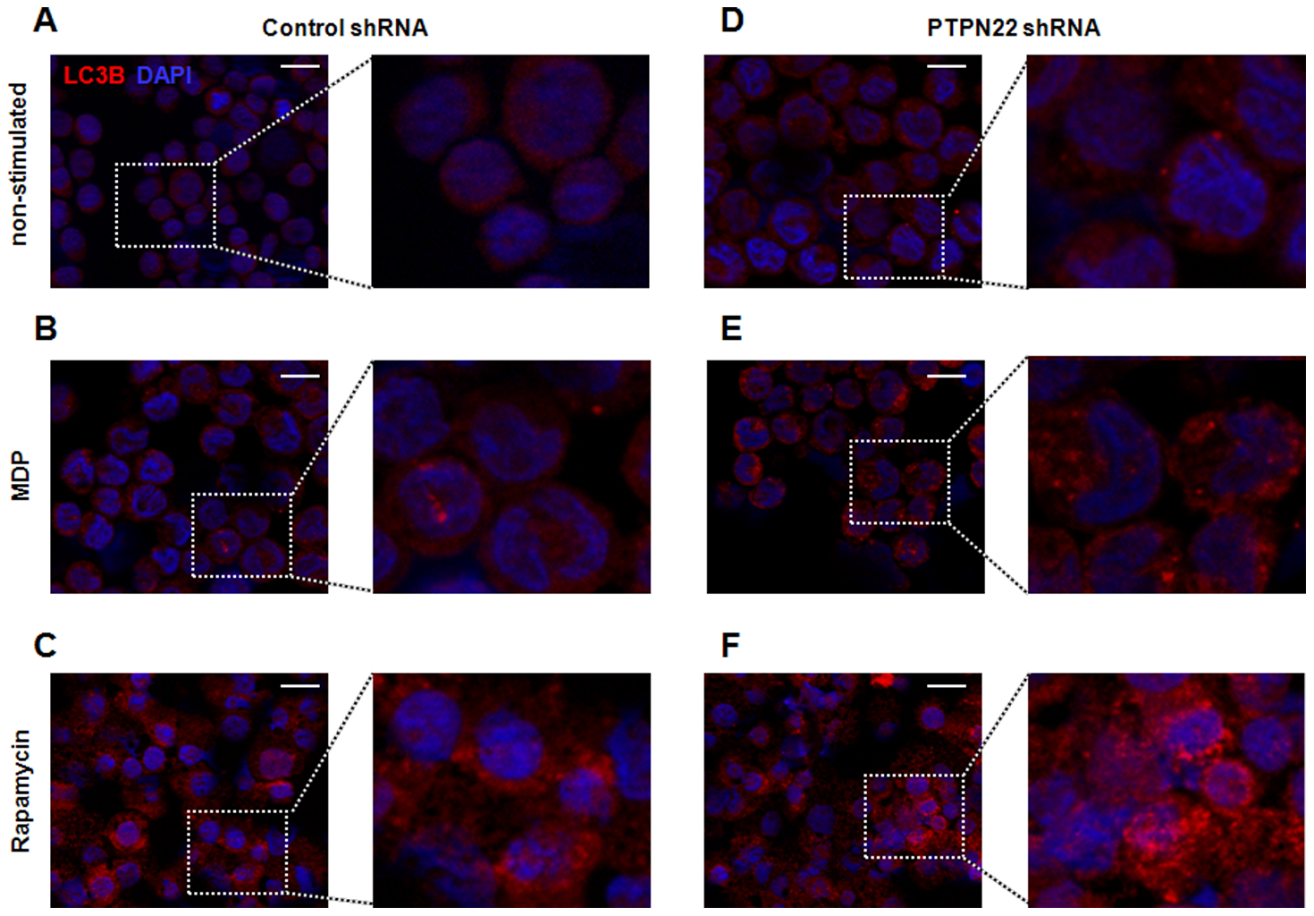
Loss of PTPN22 promotes the formation of autophagosomes. THP-1 cells, expressing either non-targeting control shRNA (**A–C**), or PTPN22 targeting shRNA (**D–F**) were left untreated, treated for 24 h with 500 ng/ml MDP or 100 nM rapamycine and stained for LC3B (red) and DAPI (blue). Representative pictures are shown for (**A**) non-treated cells expressing control shRNA, (**B**) MDP-treated cells expressing control shRNA, (**C**) Rapamycine-treated cells expressing control shRNA, (**D**) non-treated cells expressing PTPN22 shRNA, (**E**) MDP-treated cells expressing PTPN22 shRNA; and (**F**) Rapamycine-treated cells expressing PTPN22 shRNA. Scale bar: 10 µm.

To further address autophagy induction, THP-1 cells were treated for 24 h with MDP or the autophagy activator rapamycine and whole cells fluorescently stained for LC3B. In untreated, PTPN22 competent cells, LC3B staining was diffuse and rather weak (Figure 8A). When cells were stimulated with MDP or rapamycine, bright LC3B spots, indicative for autophagosome formation became visible (Figures 8B+C). Correlating to our Western blot data, in PTPN22 knockdown cells however, bright LC3B dots were already detectable in untreated cells and did further increase by MDP or rapamycine-treatment (Figures 8D–F). Together with the results from protein analysis, this indicates that loss of PTPN22 enhances autophagy.

## Discussion

Here, we demonstrate that PTPN22 is activated by MDP-treatment, and its loss interferes with signaling events downstream of NOD2 receptor in human THP-1 cells and mouse BMDC. By controlling MAPK and NF-κB activation, loss of PTPN22 influences MDP-induced gene expression, cytokine release and autophagy. We show that loss of PTPN22 results in enhanced MDP-mediated p38, JNK and NF-κB p65 phosphorylation, all involved in monocyte and macrophage differentiation and activation [21]–[24].

In IBD, hyper-activated intestinal macrophages are important drivers of intestinal inflammation [25]. Our results suggest that loss of PTPN22 renders monocytes and intestinal macrophages more reactive towards bacterial antigens, possibly leading to hyper-activation finally resulting in a chronic inflammatory state of the intestinal mucosa. Ultimately, loss of PTPN22 results in enhanced secretion of the pro-inflammatory cytokines IL-6, IL-8 and TNF, all highly increased in IBD [26], [27]. While IL-8 is involved in recruiting neutrophils to inflamed sites, and thereby enhances innate inflammatory events, IL-6 is involved in the switch from innate to adaptive immune responses [28], it activates B-cells, and plays a role in shaping the T-helper (Th) cell response [29]. Increased levels of IL-6 promote the development of IL-17 secreting T-cell subsets [29], which are found expanded in CD and play an important role in disease pathogenesis [30]. IFN-γ, on the other hand, is involved in controlling the development of Th17 cells [31] and exerts protective effects in a mouse model of acute colitis [32]. Therefore, our data suggest how loss of PTPN22 might contribute to increased secretion of pro-inflammatory mediators in the intestinal mucosa what could finally result in a chronic inflammatory state of the gastrointestinal tract establishing IBD.

Cao *et al* found decreased levels of ERK phosphorylation and enhanced levels of p38 phosphorylation in patients carrying a gain of function variant of PTPN22, indicating that PTPN22 would regulate ERK signaling and facilitate p38 activation [33]. However, there is evidence that the PTPN22 gain of function variant leads to reduced stability of the PTPN22 protein, resulting in decreased levels of PTPN22 [34]. This would be in line with our findings here, indicating that reduced levels of PTPN22 result in decreased ERK activation but enhanced p38 phosphorylation.

p38-MAPK signaling is important for shaping the monocyte/dendritic cell-induced adaptive immune reaction and its presence in antigen presenting cells is crucial for Th17 cell development [35]. The enhanced activity of p38-MAPK upon loss of PTPN22 might therefore directly influence the capacity of monocytes to promote T-cell development. In line with this, reduced PTPN22 levels result in decreased levels of the Th1 cell transcription factor T-bet, which is important for IFN-γ secretion in both, adaptive and innate immune cells [36]. Together with enhanced IL-6 and IL-8 secretion, this indicates that loss of PTPN22 could result in an altered ability of monocytes to prime specific T helper cell responses.

In line with our previous findings in IFN-γ-treated cells [16], where loss of PTPN22 decreased p65 phosphorylation, loss of PTPN22 had different effects on NF-κB activation depending on the used stimulus. NF-κB subunits are serine phosphorylated and can therefore not be a direct target of PTPN22. As we showed previously in IFN-γ-treated cells, where p65 activity is down-regulated by silencer of cytokine signaling (SOCS)3 [16], differentially activated additional regulatory pathways could mediate the observed stimulus dependent differences in NF-κB phosphorylation in MDP-treated cells.

We further observed that loss of PTPN22 results in enhanced autophagosome formation in response to MDP. Autophagy plays a crucial role in the clearance of intracellular bacteria [37], hence the enhanced autophagy, detectable in PTPN22 deficient cells, seems to provide anti-inflammatory effects on a first sight. This is surprising, as we could previously detect decreased levels of PTPN22 in intestinal biopsies from IBD patients [16]. However, autophagy is also important for the activation and differentiation of monocytes into macrophages [38] and plays a role in major histocompatibility complex (MHC)-II mediated antigen presentation [8]. In addition, JNK mediated autophagy is important to prevent activated macrophages from apoptosis [38]. Therefore, it is well possible that increased autophagy, as detectable upon loss of PTPN22, could lead to a prolonged survival of activated monocytes/macrophages. Together with enhanced pro-inflammatory cytokine secretion, this might result in an increased activation of the adaptive immune response and ultimately promote inflammatory conditions.

It has been further demonstrated that p38 and JNK activation play a role in autophagy induction in monocytic cells [38], [39]. Hence the increased p38/JNK-MAPK activity detected upon loss of PTPN22 could lead to the enhanced autophagy induction detectable in these cells. However, further experiments would be necessary to reveal the precise mechanism of how PTPN22 influences autophagy induction.

In summary, our data indicate that PTPN22, beyond its important role in regulating receptor signaling in the adaptive arm of the immune system [13], [40], also interferes with innate immune receptor pathways and innate immune functions such as autophagy. We could demonstrate that loss of PTPN22 interferes with signaling pathways induced by bacterial components and it is involved in the fine-tuning of signaling cascades downstream of NOD2 ligation. Given the importance of NOD2 activation and autophagy induction in regulating intestinal immune responses, this might explain the association of PTPN22 variants with CD and UC.

## Supporting Information

Figure S1 **Different effect of distinct bacterial products on PTPN22 mRNA expression.** THP-1 cells were treated for increasing time with **(A)** 100 ng/ml LPS, **(B)** 100 ng/ml PamCys, or **(C)** 100 ng/ml C12-iE-DAP. Graphs show PTPN22 mRNA expression relative to non-treated control and normalized to β-actin. Asterisks denote significant differences from the non-treated control (n = 3 each, * = p<0.05, ** = p<0.01, *** = p<0.001).
(TIF)

Figure S2 **PTPN22 controls p38 MAPK.** **(A+B)** THP-1 cells were treated for the indicated time with 500 ng/ml MDP. Representative Western blots show levels of **(A)** phospho-p38 (Thr^180^/Tyr^182^) and total p38; and **(B)** phospho-ERK (Tyr^42^/Tyr^44^) and total ERK. **(C–E)** THP-1 cells were treated for 30 min with **(C)** LPS, **(D)** PamCys, or **(E)** C12-iE-DAP. Representative Western blots show levels of phospho-p38 (Thr^180^/Tyr^182^) and total p38 and phospho-ERK (Tyr^42^/Tyr^44^) and total ERK. Asterisks denote significant differences from the non-treated control (n = 3 each, * = p<0.05, *** = p<0.001); # = p<0.05 *vs.* MDP treatment of control-transduced THP-1 cells.
(TIF)

Figure S3 **Loss of PTPN22 affects NF-κB in a stimulus dependent manner.** **(A+B)** THP-1 cells were treated for the indicated time with 500 ng/ml MDP. Representative Western blots show levels of **(A)** phospho-NF-κB p65 (Ser^536^) and total NK-κB p65; and **(B)** phospho-NF-κB p105 (Ser^933^) and total NF-κB p105. **(C+D)** THP-1 cells were treated for 30 min with 500 ng/ml MDP. Representative Western blots and densitometric analysis show levels of **(C)** phospho-NF-κB p100 (Ser^866^/Ser^870^) and total NF-κB p100; and **(D)** phospho-NF-κB p52 (Ser^933^) and total NF-κB p52. **(E–G)** THP-1 cells were treated for 30 min with **(E)** LPS, **(F)** PamCys or **(G)** C12-iE-DAP. Representative Western blots show levels of phospho-NF-κB p65 (Ser^536^) and total NK-κB p65 and of phospho-NF-κB p105 (Ser^933^) and total NF-κB p105. Asterisks denote significant differences from the non-treated control (n = 3 each, *** = p<0.001).
(TIF)
