# Supplementary figures and images for: Protein Tyrosine Phosphatase Non-Receptor Type 22 Modulates NOD2-Induced Cytokine Release and Autophagy
Source: PLoS One. 2013 Aug 26;8(8):e72384. doi: 10.1371/journal.pone.0072384 (PMC3753240; doi:10.1371/journal.pone.0072384)

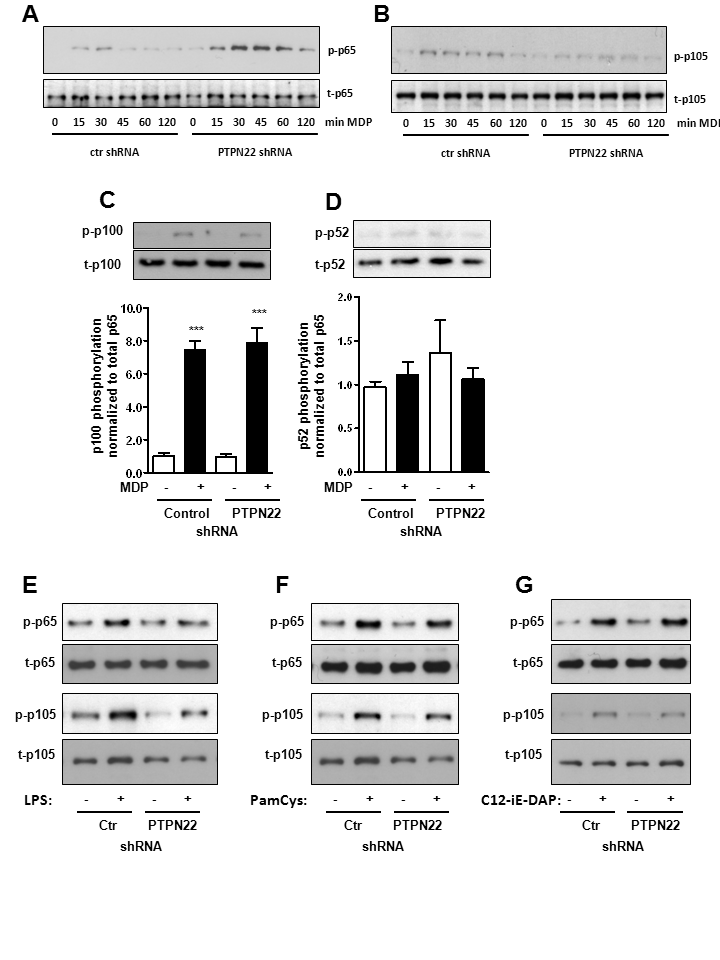

Supplement: Figure S3 — Loss of PTPN22 affects NF-κB in a stimulus dependent manner. (A+B) THP-1 cells were treated for the indicated time with 500 ng/ml MDP. Representative Western blots show levels of (A) phospho-NF-κB p65 (Ser536) and total NK-κB p65; and (B) phospho-NF-κB p105 (Ser933) and total NF-κB p105. (C+D) THP-1 cells were treated for 30 min with 500 ng/ml MDP. Representative Western blots and densitometric analysis show levels of (C) phospho-NF-κB p100 (Ser866/Ser870) and total NF-κB p100; and (D) phospho-NF-κB p52 (Ser933) and total NF-κB p52. (E–G) THP-1 cells were treated for 30 min with (E) LPS, (F) PamCys or (G) C12-iE-DAP. Representative Western blots show levels of phospho-NF-κB p65 (Ser536) and total NK-κB p65 and of phospho-NF-κB p105 (Ser933) and total NF-κB p105. Asterisks denote significant differences from the non-treated control (n = 3 each, *** = p<0.001). (TIF) [file pone.0072384.s003.tif]
